# Supplementary material for: Causal relationships between gut microbiome and hundreds of age-related traits: evidence of a replicable effect on ApoM protein levels
Source: Aging (Albany NY). 2025 Aug 1;17(8):1966–87. doi: 10.18632/aging.206293 (PMC12422825; doi:10.18632/aging.206293)
Supplement: Supplementary Figures [file aging-17-8-206293-s001.pdf]

SUPPLEMENTARY FIGURES

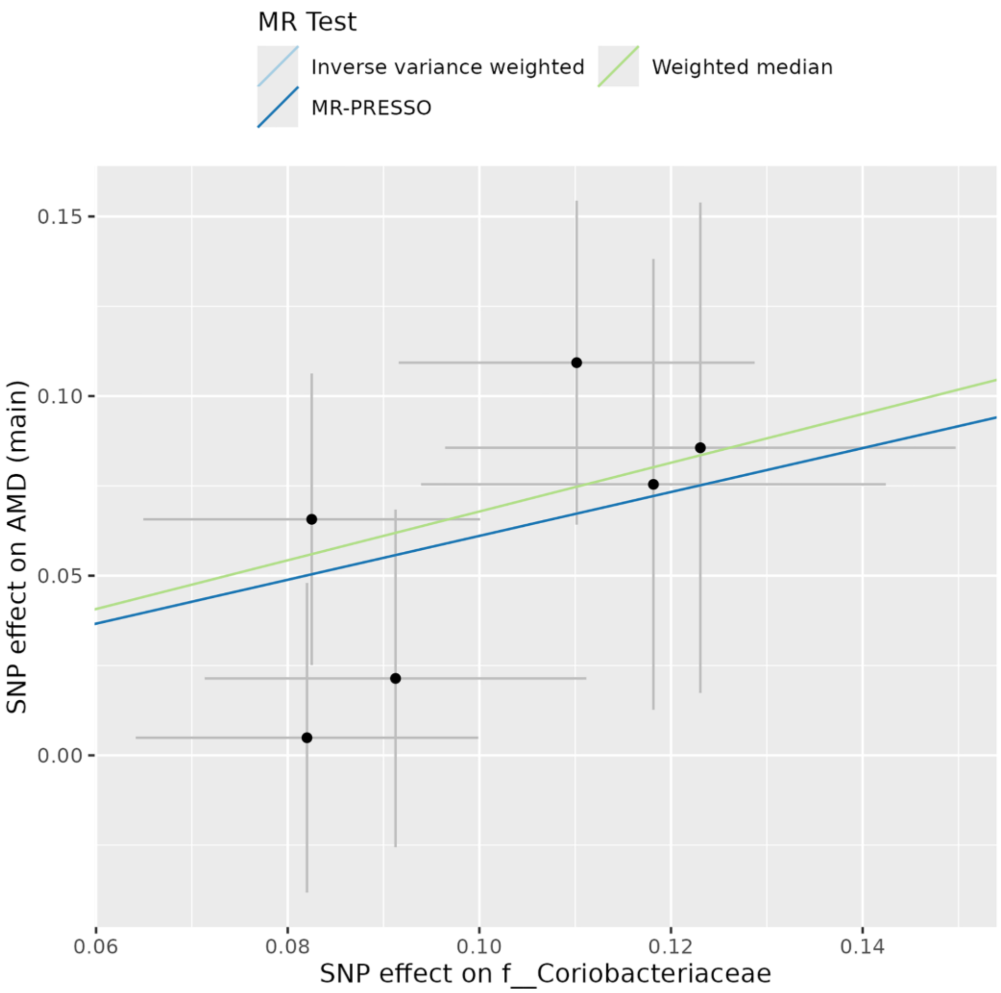

Supplementary Figure 1. Scatter plot of main MR analysis between Coriobacteriales and age-related macular degeneration. This figure shows the results of the main MR analysis between Coriobacteriales (o__Coriobacteriales or f__Coriobacteriaceae) and AMD. In the scatter plot, each dot is an IV and the x and y-axis represents the association coefficients with the exposure and outcome, respectively. The three lines represent the results of the three different MR tests, with the slope of the lines being equal to causal estimates of each test. Abbreviations: AMD: age-related macular degeneration; IV: instrumental variable; MR: Mendelian randomization; SNP: single nucleotide polymorphism.

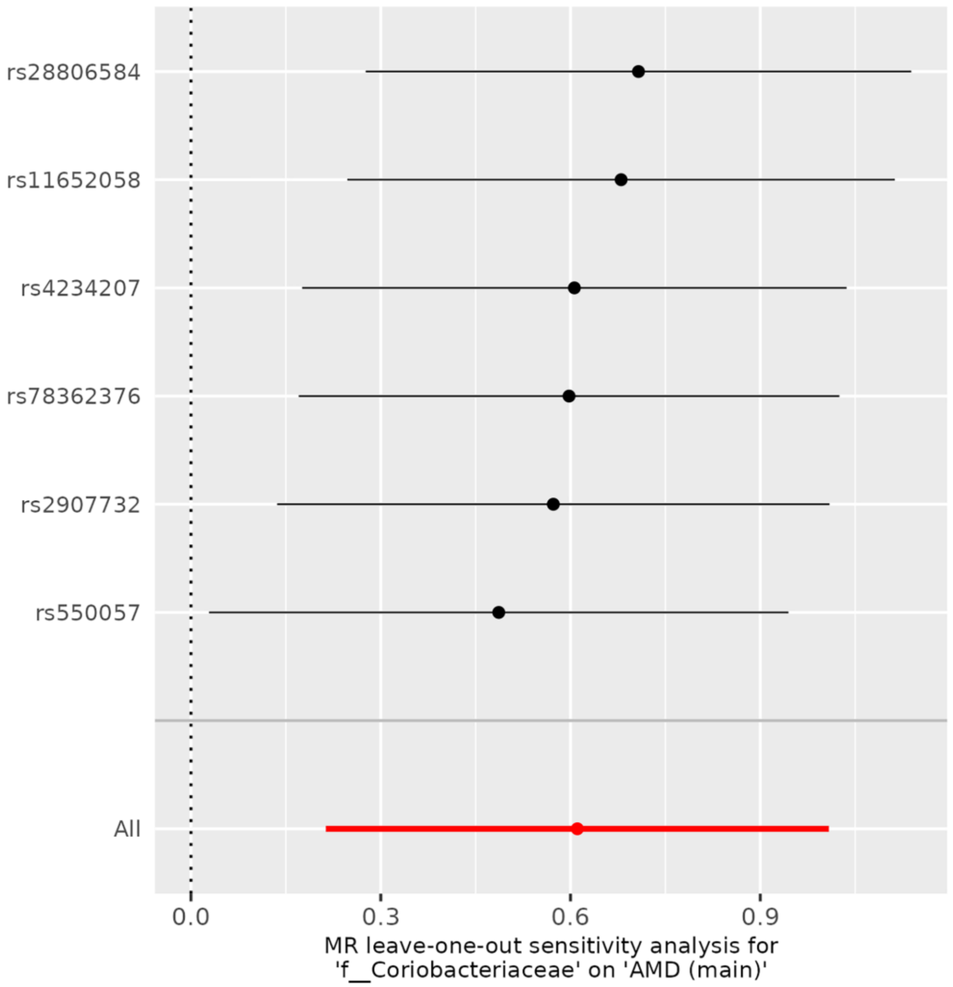

Supplementary Figure 2. Leave-one-out plot of the main MR analysis between *Coriobacteriales* and age-related macular degeneration. In this figure the causal estimates from the leave-one-out analyses between *Coriobacteriales* (*o__Coriobacteriales* or *f__Coriobacteriaceae*) and AMD are shown and compared with the causal estimate from main analysis (red line). Abbreviations: AMD: age-related macular degeneration; MR: Mendelian randomization.

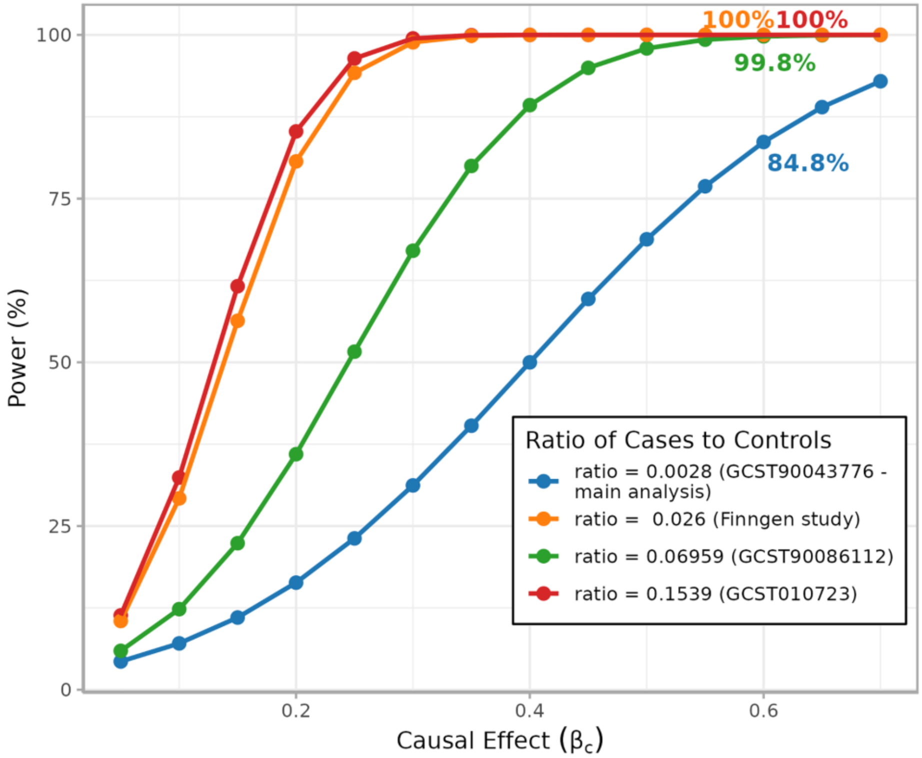

Supplementary Figure 3. Power estimates of *Coriobacteriales* vs age-related macular degeneration analysis. This plot shows the power estimates at varying causal effect sizes β_c, for independent studies of age-related macular degeneration as outcome. The causal effect identified in the main analysis (β_c = 0.061) and corresponding power are highlighted in the curves.

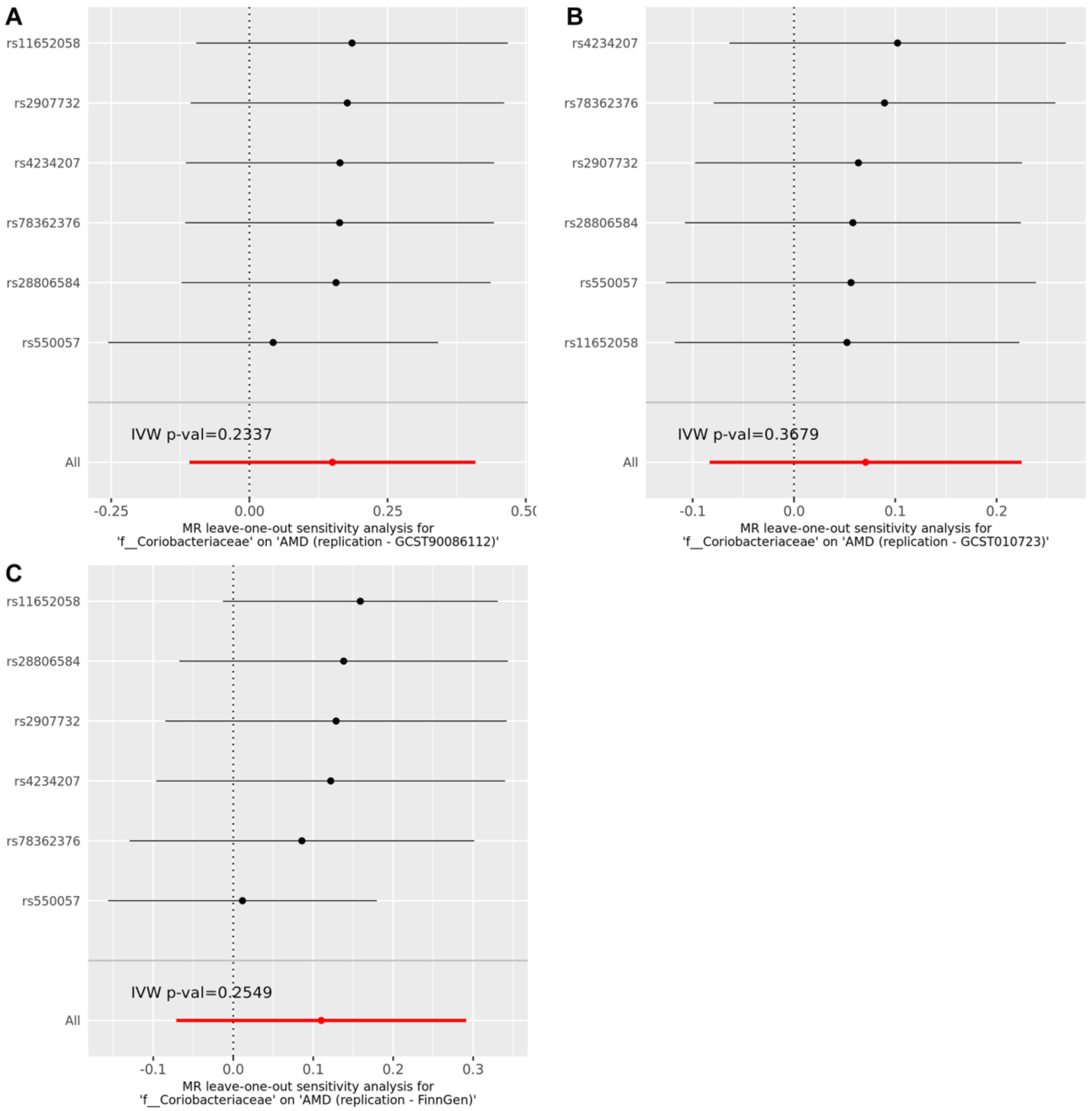

Supplementary Figure 4. Replication analyses between *Coriobacteriales* vs. independent GWASs of age-related macular degeneration. The (**A**) shows the leave-one-out plot of MR replication with GWAS of age-related macular degeneration (AMD2) from Guindo-Martinez et al., 2021 (GCST90086112) and the exposure *Coriobacteriales* (*o__Coriobacteriales* or *f__Coriobacteriaceae*). The (**B**) shows the leave-one-out plot of MR replication analysis with "Early AMD" GWAS from Winkler et al., 2020 (GCST010723) as outcome. The (**C**) represents leave-one-out plot of replication analysis with AMD GWAS from FinnGen study (https://www.finngen.fi/en/access_results) as outcome. Abbreviations: AMD: age-related macular degeneration; GWAS: genome-wide association study; IVW: inverse variance weighted; MR: Mendelian randomization.

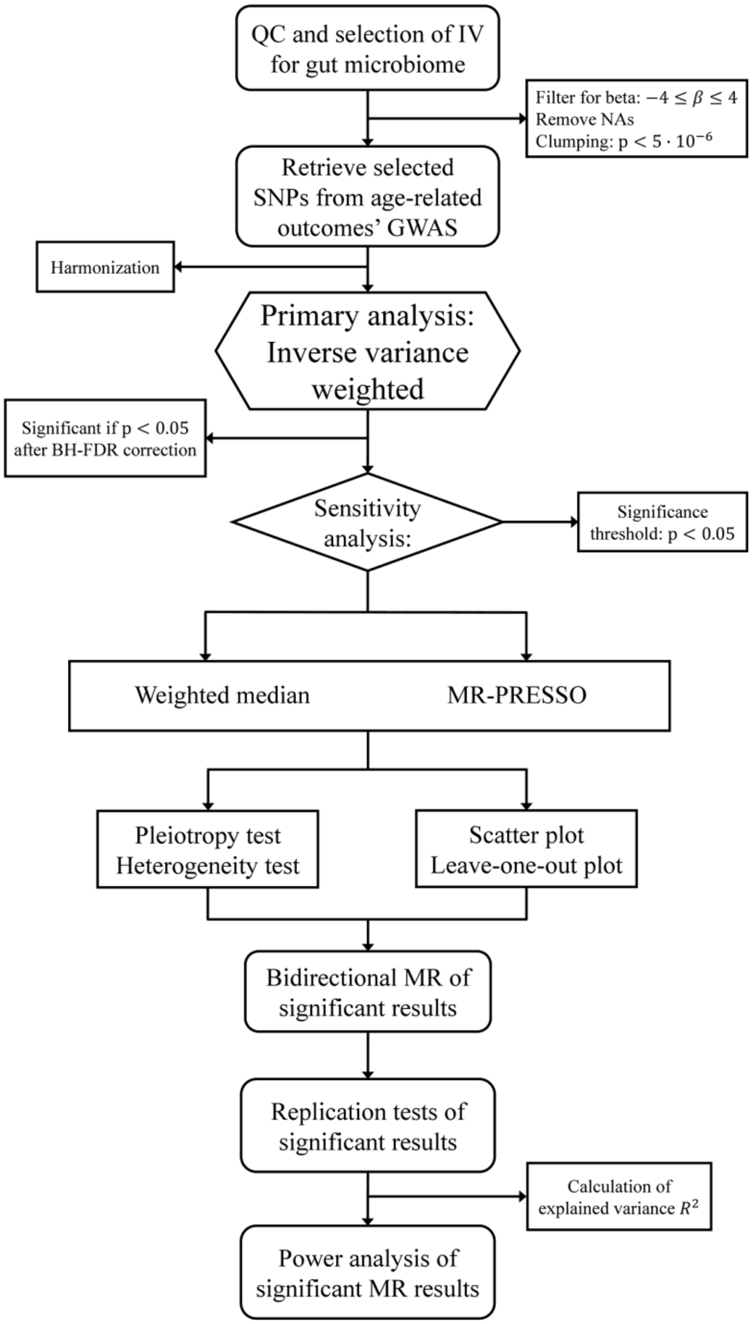

Supplementary Figure 5. Workflow of the study. This workflow shows all the steps of the MR analysis examining the causal link between the gut microbiome and specific age-related outcomes. Abbreviations: IV: instrumental variable; GWAS: genome-wide association study; MR: Mendelian randomization; NAs: missing values; SNP: single nucleotide polymorphism; QC: quality control.
